# Implementing frailty interventions in hospitals: A systematic review of strategies and outcomes

**DOI:** 10.1111/ajag.70060

**Published:** 2025-06-24

**Authors:** Kisani Manuel, Madison Chapman, Maria Crotty, Gill Harvey, Susan E. Kurrle, Kate Laver

**Affiliations:** ^1^ Rehabilitation, Aged and Palliative Care Division Southern Adelaide Local Health Network Bedford Park South Australia Australia; ^2^ Flinders Health and Medical Research Institute, College of Medicine and Public Health Flinders University Adelaide South Australia Australia; ^3^ Caring Futures Institute, College of Nursing and Health Sciences Flinders University Adelaide South Australia Australia; ^4^ Faculty of Medicine and Health University of Sydney Sydney New South Wales Australia; ^5^ Rehabilitation and Aged Care Services Northern Sydney Local Health District Sydney New South Wales Australia

**Keywords:** frailty, geriatric nursing, hospitals, implementation science, knowledge translation, quality improvement

## Abstract

**Objectives:**

This systematic review aimed to identify the nature and effects of implementation strategies used to improve the care of older people with frailty in hospital settings.

**Methods:**

We included randomised controlled trials (RCTs), non‐RCTs, before–after studies and interrupted time series describing clinical frailty‐focussed interventions and implementation strategies aimed at improving outcomes for older people with frailty in hospital settings. We included peer‐review articles and PhD theses published from the Year 2000 onwards. We excluded publications not in English and conference abstracts. Four electronic databases (Medline, PsycInfo, CINAHL and Scopus) were searched, alongside grey literature, in April 2024. Risk of bias was analysed using the NIH Quality Assessment Tool. A narrative synthesis approach was undertaken, with the RE‐AIM framework used to present data for implementation outcomes and the Expert Recommendations for Implementing Change (ERIC) taxonomy used to categorise implementation strategies.

**Results:**

Fifteen studies were included; all were pre‐/postdesigns and published post‐2014. Most studies involved implementing frailty assessments to trigger care planning and pathways for people with frailty. Twelve studies reported positive improvements in one or more primary outcomes. Common implementation strategies included developing quality monitoring tools, mandating change, promoting adaptability of the intervention and distributing educational materials.

**Conclusions:**

Frailty interventions in hospital settings are usually multicomponent and highly influenced by context. This review confirms the feasibility of frailty screening and intervention in hospital settings, but implementation strategies are not well‐reported. Future research should prioritise rigorous study designs and reporting to optimise the transferability of successful implementation strategies for frailty interventions to other health‐care settings.

**Practice impact**

Regardless of the frailty screening tool used, all studies showed an improvement in screening and diagnosis rates, indicating the feasibility and utility of frailty screening. The impact of frailty interventions on other outcomes is less clear, highlighting a need for more research, particularly considering the impact on quality of life for patients.

## 1 INTRODUCTION

Frailty is a dynamic state of heightened vulnerability to stressors.^1^ It is a common condition in hospitalised older adults with an estimated prevalence of 27%–80%.^2^ Frailty is significantly associated with poorer outcomes, including increased mortality and hospital length of stay.^3^, ^4^ The global burden of frailty has considerable implications for health systems, social care infrastructure and economic sustainability, particularly as the proportion of older adults continues to grow. Addressing frailty is essential for promoting healthy ageing, reducing health‐care utilisation and achieving broader public health goals such as those outlined in the World Health Organization's *Decade of Healthy Ageing* initiative.

Clear guidelines are available to support the identification and management of frailty. The Asia‐Pacific Clinical Practice Guidelines strongly recommend (1) using a validated measurement tool to identify frailty, (2) prescription of physical activity with a resistance component and (3) addressing polypharmacy. They also recommend screening for and addressing modifiable causes of fatigue, addressing weight loss and prescribing vitamin D for people who are deficient.^5^ In addition, the International Conference on Frailty and Sarcopenia Research (ICFSR) International Clinical Practice Guidelines for Identification and Management of Physical Frailty include a recommendation for social support and encourage a comprehensive care plan.^6^

Despite the availability of these guidelines, a gap exists between recommendations and clinical practice.^7^ Frailty often remains underdiagnosed and undertreated, especially in low‐resource settings. Reasons for this are likely multifactorial and may include a lack of training on frailty, lack of hospital protocols, limited consensus on frailty assessment and screening tools and resource constraints.^8^, ^9^

A recent systematic review on community‐based frailty management identified resource distribution, patient engagement and professional skill sets as key factors for translating frailty interventions into practice.^10^ However, less is known about how frailty assessment and management are implemented in hospital settings, where much of health care for older people occurs.^11^ Frailty is very common among hospitalised older people, and hospitalisation in itself can increase the risk of developing or worsening frailty.^12^

Clinical trials in hospital settings have shown that interventions such as exercise, nutritional supplementation and comprehensive geriatric assessment (CGA) can effectively improve frailty outcomes in hospital.^13^, ^14^ However, translating these interventions into routine practice remains challenging, as health‐care systems generally struggle to implement research findings.^15^, ^16^, ^17^ Gaining insight into (1) how frailty interventions are implemented in real‐world settings and (2) the outcomes they achieve will help advance knowledge in this field.

The aim of this review was to systematically examine studies that have implemented frailty management interventions in hospital settings. This will provide a better understanding of the implementation strategies used, the outcomes achieved and the challenges encountered. By identifying effective approaches and common barriers, this review sought to inform future efforts to translate frailty interventions into routine hospital care and ultimately improve outcomes for older adults with frailty.

## 2 METHODS

This was a systematic review of implementation studies meeting the inclusion criteria below, with a focus on implementation strategies used within the studies. The protocol was designed a priori, and the review was registered with PROSPERO (PROSPERO 2022 CRD42022360172). The reporting of this systematic review adheres to the Preferred Reporting Items for Systematic Reviews and Meta‐Analyses (PRISMA) (see Data S1).

### 2.1 Inclusion and exclusion criteria

Studies were included if they involved a randomised controlled trial (RCT), non‐RCT, before–after study or interrupted time‐series design (where at least three time points were available before and after intervention) that described an intervention and implementation strategies aimed at improving outcomes (all outcomes considered) for older people with frailty. Implementation strategies are defined as ‘a method or technique designed to enhance adoption of a clinical intervention’.^18^ The intervention needed to be administered in the hospital setting (inpatient, outpatient and clinics) and the population had to be classified as prefrail or frail based on assessment with an accepted measurement of frailty. The Asia‐Pacific Clinical Practice Guidelines for the Management of Frailty outline that measures of frailty should accurately detect frailty, be well‐validated, simple to use, predictive of patient outcomes and clinically appropriate to the given setting.^5^ Studies that only identified frailty subjectively through superficial visual appearance were excluded in alignment with guideline recommendations.^5^ We included papers published from 2000 to March 2023 (based on emergence of frailty literature around that time) and PhD theses. We excluded papers not published in English (as we had no translation funding available) and conference abstracts.

### 2.2 Search strategy

We searched four electronic databases (Medline, PsycInfo, CINAHL and Scopus) in April 2024. The search strategy for Medline is presented in Data S2 and was adapted for other databases with assistance from a medical librarian. We also searched grey literature using a Google scholar search, where we reviewed the first 200 citations.

One review author (removed for peer review) did the initial search. Two authors independently screened titles and abstracts for each study against the eligibility criteria using Covidence software (removed for peer review). Upon completion of screening, the two review authors independently read the full text of potentially eligible studies to determine eligibility. Any disagreements were settled by discussion between the two authors.

### 2.3 Data extraction

Two authors (removed for peer review) extracted data from the included studies independently into three separate tables. Discrepancies in extraction were resolved through discussion between reviewers. Data extracted into Table 1 included a summary of the included studies, details of the clinical intervention, study design and strategy, use of theory, practice change and clinical outcomes. Data extracted into Table 2 included details of the implementation strategies used in each study, reported according to the Expert Recommendations for Implementing Change (ERIC) categories.^19^ The ERIC taxonomy provided a comprehensive and structured framework allowing reviewers to map and group strategies to identify commonly used, or underutilised, strategies. Data extracted into Table 3 included implementation outcomes displayed in the form of Reach, Effectiveness, Adoption, Implementation and Maintenance (RE‐AIM) framework domains.^20^ The RE‐AIM framework was chosen because it provides a useful structure for understanding issues related to the design, dissemination and implementation of interventions in health‐care settings, and it was used to guide the data synthesis process.^20^

**TABLE 1:** Characteristics of included studies.

First author (year) and country	Clinical intervention being implemented	Study design of implementation strategy	Nature of implementation strategy	Use of theory or other approaches to select implementation strategies	Practice change/Adoption (number providing intervention)	Clinical outcomes
Bakker (2014) The Netherlands	*Content:* CareWell in Hospital (CWH) program includes screening for frailty and tailored intervention in hospital. Intervention includes a CareWell management plan, medication review, comprehensive geriatric assessment, discussion at multi‐disciplinary meetings, and physical and cognitive activities *Duration:* Length of inpatient hospital admission *Delivered by:* Geriatrician and geriatric nurse, department nurses and physicians, trained volunteers	Quasi‐experimental pre‐/poststudy with a process evaluation	Development of specific proactive CareWell team Volunteers trained and continuous education provided to physicians and nurses Meetings with professionals within departments were organised during implementation period to enhance and monitor the progress and integration of program Process evaluation by monitoring intervention fidelity to enhance effectiveness of implementation	Based on the Hospital Elder Life Program (HELP) comprising 2 main concepts: Proactive and intensive support by a CareWell geriatrics team to improve patient‐centred care for older patients with frailty and increased awareness and competency among nurses and physicians with respect to providing geriatric care The introduction of a team of trained volunteers to offer activities for timely cognitive and physical stimulation to patients and to integrate care and well‐being‐directed activities within the hospital	Number of organisations: Three departments in one hospital in the Netherlands, total of 116 beds across the departments Number of interventionists: Not specified. Multiple	Primary outcomes: * Incidence of hospital‐acquired delirium * Cognitive decline * Decline in ADLs during hospital admission Secondary outcomes: * Unplanned readmission within 1 month of discharge * Change in ADL function at 3 months There was no significant difference detected between pre‐CWH and post‐CWH groups There was decreased caregiver self‐rated burden of care in the Post‐CWH group (*p* = .049) There was improved cognitive functioning at discharge for surgical patients, as well as improved ADL functioning
Bryant (2019) United States	*Content:* An interdisciplinary protocol for frail trauma patients called the ‘The Frailty Identification and Care Pathway’ Included screening for frailty, a standardised order set, consultants by geriatrics, physical therapy, nutrition and social work within 72 h, a multid family meeting for patients with hospital stay ≥5 days, fall prevention education *Duration:* Length of inpatient hospital admission *Delivered By:* Geriatrician, resident doctors, nurses (including a nurse champion), multidisciplinary team	Quasi‐experimental pre/post	Educational sessions for multidisciplinary staff. A pocket card for doctors. A nursing champion. Peer Education. A paper checklist. An online mandatory educational module for completion prior to joining the trauma service. Biweekly rounds of trauma unit and ICU to reinforce pathway principles and allow trauma geriatrician to directly answer staff questions	Educational sessions for multidisciplinary staff. A pocket card for doctors. A nursing champion. Peer Education. A paper checklist. An online mandatory educational module for completion prior to joining the trauma service	Number of organisations = 1 Number of interventionists: Not specified. Multiple	Primary outcomes: incidence of delirium, major complications during admission, mortality and 30‐day readmission There was a significant decrease in delirium (22%–13%, respectively, *p* = .04) and 30‐day readmission (10%–3%, *p* = .01) There were no significant differences in complications between pre‐ and postintervention cohorts (28% vs. 29%, *p* = .93), or in‐hospital mortality (7% vs. 4%, *p* = .28)
Chen (2014) Taiwan	*Content:* Modified HELP Interventions (mHELP) comprising of three interventions. (1) Early mobilisation (2) Oral and nutritional assistance (3) Orientation communication HELP nurse provided three interventions three times daily, adding 30–45 min per day of care to each participant Included mobilisation intervention, communication, oral care, and diet education *Duration:* Length of hospital admission (range 4–20 days) *Delivered By:* A registered nurse trained as the ‘HELP nurse’	Quasi‐experimental pre/post	Used standardised mHELP manuals for training the HELP nurse Weekly individual mentorship for HELP nurse Outcome assessors trained and underwent performance checks every 3 months to avoid deviation from measurement protocol	Adaptation of the Hospital Elder Life Program (HELP) to focus on components that address shared risk factors of cognitive, functional, and nutritional status to fit a surgical setting in Taiwan Modification based on the theory that frailty might be managed by the same intervention as other geriatric syndromes because of shared risk factors	Number of organisations = 1 ward (gastrointestinal ward) with 36 beds. Located in an urban hospital in Taiwan Number of interventionists: 1 registered nurse	Primary outcomes: Frailty rates and transitions between frailty states from hospital discharge to 3 months after discharge Frailty rate at hospital discharge was reduced (19% of intervention group vs. 65% of control group, *p* < .001). Transitions between frailty states also differed significantly between the groups 3 months after hospital discharge there was no significant difference between the rate of frailty between the intervention and control group (17% vs. 23%, *p* = .62)
Ehrlich (2023) United States	*Content:* Implemented a geriatric surgery pathway. Patients 65 and older who were considered high‐risk for frailty based on Edmonton Frail Scale were referred for Comprehensive Geriatric Assessment and discussed on multidisciplinary preoperative call. Patients on pathway seen and/or followed by Geriatric Medicine, providing guidance on medication management and delirium prevention. Nutritional services provided as well as mobility program *Duration:* Duration of hospital admission *Delivered by:* Multidisciplinary team	Pre‐/postimplementation study	Implementing pathway for patients identified as high‐risk for frailty Nursing staff underwent online geriatric resource training through Nurses Improving Care for our Health System Elderly (NICHE) program NICHE program includes education on medication, delirium screening and prevention, and ambulation	Pathway implemented in compliance with 32 evidence‐based standards of the American College of Surgeons' Geriatric Surgery Verification Program (ACS‐GSV). Standards reflect the 4 Ms (Mentation, Mobility, Medication, Matters)	Number of organisations: 1 hospital (The Johns Hopkins Bayview Medical Center: General Surgery, Orthopedics, Vascular Surgery, Plastic and Reconstructive Surgery, and Urology) Number of interventionists: Not specified	Primary outcomes: On adjusted multivariable analysis, patients attending hospital once GSP pathway had been implemented had significantly decreased risk for postoperative loss of independence and major complications than patients who attended hospital prior to pathway. GSP cohort had decreased risk for loss of independence by 46% and for major complications by 19% On adjusted multivariable analysis, GSP cohort was not associated with improvement in length of stay or readmission rate Sub‐group analysis of patients with frailty who were part of GSP cohort had significantly decreased risk for loss of independence, major complications, and reduced length of stay. There was no significant different in readmission rates
Engelhardt (2018) United States	*Content:* Development and implementation of a frailty pathway *Duration:* Duration of hospital admission with 7 days follow‐up post discharge *Delivered By:* Hospitalist (doctor) and multidisciplinary team (including physiotherapy, occupational therapy, social work)	Quasi‐experimental pre/post	* Multidisciplinary team of key stakeholders reviewed current practice, guidelines, and recommendations * Team then followed define, measure, analyse, improve, control (DMAIC) process improvement methodology to design a frailty pathway * Screening of patients for frailty * Patients with frailty received (1) Hospitalist consult, (2) Early family engagement (3) Palliative care consultant when appropriate (4) expediated SW and PT/OT evaluation * Use of specialised order set * Frailty follow‐up: 3‐day phone call after discharge, 7‐day follow‐up appointment after discharge	Designed and implemented as a QI project following DMAIC principles	Number of organisations: 1 hospital (Northwest Memorial Hospital in Chicago, Illinois) Number of interventionists: Not specified. Multiple	Primary outcomes: Length of stay, Loss of Independence, 30‐day readmission rate Reduction in length of stay (9–6 days) (*p* = .4), readmissions decreased from 36% to 10% (*p* = .02), and LOI (loss of independence) decreased by 40% (100% vs. 60%; *p* = .01)
Ernst (2014) United States	*Content:* Developed screening tool called risk analysis index (RAI). Implemented formal screening process to identify patients with frailty considering elective surgery For patients from 1 January 2006 to 31 August 2006 determined palliative care consults and retrospectively from medical records determined RAI score. RAI score calculated prospectively from when screening implemented through patient interviews *Duration:* Screening at surgical appointment or on admission to hospital *Delivered By:* Nurses, clinicians, medical assistants	Quasi‐experimental pre/post	Developed and validated frailty screening tool (RAI). Determined RAI's ability to discriminate patients with or without frailty using data from National Surgery Quality Improvement Project and local databases Mandated that all elective surgery patients were screened Review of patient notes by the chief of surgery or designee. Frail patients were encouraged to accept palliative care consultation Template consultation note was completed by Palliative Care team	Not specified	Number of organisations: Nebraska Western Iowa Veterans Affairs Medical Center Number of interventionists: Not specified	Primary outcomes: 30‐, 180‐ and 360‐day mortality Significant increase in palliative care consult requests Significant decrease in mortality rate amongst patients with palliative care consult at 30 days (32% pre and 21% post), 180 days (71% pre and 44% post), and 360 days (79% pre and 66% post) Significantly more patients did not have surgery post implementation (6% pre and 19% post) Mean survival days not significantly different (295 pre and 314 post) Implementation of the screening program was associated with a significantly reduced odds of death
Fritsche (2023) Netherlands	*Content:* Frailty assessed retrospectively with Dutch Health and Youth Care Inspectorate (VMS) program or with Clinical Frailty Scale (CFS) for postimplementation assessment. Patient communications between health‐care providers were checked for mentioning of frailty, medication list, and resuscitation orders. Timely discharge letters were also checked *Duration:* Not specified *Delivered by:* Health‐care providers including physicians in hospital and general practitioners	Pre‐/postimplementation study	Mobile phone app created to improve communications and make it simpler to find agreements and contact information for care providers in the region Agreement developed by working group of health‐care providers for older people and includes requirements for correspondence on information including frailty, medical history, medication, and resuscitation orders. GPs expected to identify and record frailty and emergency physicians instructed to consider information provided by GPs Posters, a video, and instruction handbook for new doctors were developed. Guidelines presented during visits to departments. GPs informed through newsletters, video, mouse pads, and presentation	Guidelines for information transfer between health‐care services are outlined in regional transmural agreements. Aim to assess the success of the agreement for information transfer between general practitioners, hospitals, and care physicians concerning older people with frailty	Number of organisations: Spaarne Gasthius Hospital (200 bed teaching hospital, all departments) Number of interventionalists: Not specified	Primary outcomes: Before implementation of the agreement, frailty was mentioned in 13% of letters from the hospital compared to 15% of letters postimplementation (not significant) Geriatrics department mentioned frailty most frequently (60% of preimplementation letters and 66% of postimplementation letters) There was no significant difference in mentions of medication lists or resuscitation orders before and after implementation Number of referral letters from GP improved significantly (32%–54%). Mentions of frailty in referral letters was not significantly different before and after implementation Secondary outcomes: No significant differences in discharge letters being sent within 24 h before and after implementation (57%–61%)
Hall (2017) United States	*Content:* Screened patients for frailty using the Risk Analysis Index (RAI). Surgical quality nurse flagged patients with RAI score ≥21. Identified patients could have appropriate preoperative planning that would assist in decreasing adverse effects of procedure Where appropriate referred for formal preoperative palliative care consult *Duration:* Intervention occurred during hospital admission preoperatively Intervention delivered 1 October 2007 to 1 July 2014 *Delivered by:* Nurses and clinicians from surgical, anaesthesiology, palliative care	Quasi‐experimental pre/post	Used AORN Research Evidence Appraisal Tool – Study to appraise evidence in this study	Nebraska‐Western Iowa Health Care System chief of surgery designed and implemented quality improvement project called Frailty Screening Initiative to improve postoperative survival due to increasing postoperative mortality Informed by the Standards for Quality Improvement reporting Excellence 2 guidelines for reporting QI projects	Number of organisations: The Surgical Service Line at the Veterans Affairs Nebraska‐Western Iowa Health Care System in Omaha, Nebraska Number of interventionalists: Not specified	Primary outcomes: Postoperative mortality at 30, 180 and 365 days Quality improvement to examine facility wide preoperative screening for frailty and postoperative outcomes Compared outcomes pre and post Frailty Screening Initiative for effect on postoperative mortality 30‐day mortality significantly reduced from 2% (84 of 5275 patients before screening) to 1% (26 of 3878 patients with screening). Improvement greatest among patients with frailty: 12% (24/197) to 4% (16/424) (mortality also decreased for patients without frailty)
Hall (2022) UK	*Content:* The Specialised Clinical Frailty Network (SCFN) supported hospitals to re‐design services according to principles for supporting people with frailty SCFN program involved understanding local context, planning, discussing barriers, mapping patient pathways, feeding back recommendations to sites, supported to implement changes *Duration:* SCFN program ran for 6–9 months *Delivered By:* Implementation experts and multidisciplinary clinical teams	Pre‐/postquality improvement	Network supported by stakeholders. Used plan‐do‐study‐act cycles. Clinical and improvement experts involved. Built upon findings from the Acute Frailty Network (related but separate network)	SCFN supported by national stakeholders including NHS England, the British Geriatrics Society, the Royal College of Physicians, and other societies representing specialities Used the Model for Improvement as the quality improvement method with plan‐do‐study‐act cycles Supported by national clinical and improvement experts	Number of organisations: 50 clinical teams (number of hospitals not specified, specialities including nephrology, oncology, cardiac surgery) Number of interventionalists: Not specified	Primary outcomes: 41 teams implemented frailty assessment into pathways of care 28% of teams had mechanisms for early identification of people with frailty pre‐SCFN compared to 95% of teams following SCFN There was an increase in percentage of teams adhering to every SCFN principle Barts Health NHS Trust transcatheter aortic valve implantation (TAVI) service reduced average length of stay from 3 to 2 days by having different pathways for patients with different levels of frailty Secondary outcomes: 24 of the first 28 teams to participate in network agreed that their understanding of QI tools and techniques had improved
Heim (2016) The Netherlands	*Content:* Transitional care program involving development, implementation, and evaluation of host of innovations Patients admitted to hospitals aged 70 years and older were interviewed by nurse. Patients with frailty would have need for individualised care path assessed Innovations differed between hospitals but included based on four areas; (1) risk management, (2) integrated, multidisciplinary function‐oriented care, (3) specific geriatric interventions, (4) optimisation of transfers between centres *Duration:* Duration of hospital admission *Delivered By:* Range of health professionals including nurses and doctors	Quasi‐experimental pre/post	Organised meetings with stakeholders across the region to seek commitment to the program, agenda and main objectives co‐created in meetings Committees including older adults established to identify deficiencies in organisations and collaborations and propose innovations to work towards objectives Proposed innovations initiated by a project manager from organisations involved, region‐wide innovations initiated by project team Strategies across innovations included: Protocols and guidelines created. Education for clinical teams. Multidisciplinary weekly meetings. Development of dashboard to visualise whether indicators were meeting the norm. Triage instrument and website developed for ease of use. Multidisciplinary team input for decisions around transferring patients. Implementation and optimisation of digital health system	The National Care for the Elderly Program started in the Netherlands to improve health care for older adults. As part of national program, the transitional care program was implemented in region of Netherlands	Number of organisations: 4 hospitals and other health‐care providers Number of interventionists: Not specified. Multiple	Primary outcomes: Pre and post adverse outcomes 3 months after admission to hospital Pre and post costs of long‐term care In patients with frailty, the incidence of adverse outcomes decreased from 49% (149/303) in the preprogram sample to 36% (130/366) in the postprogram sample Overall, there was a non‐significant mean different in cost favouring the preprogram sample
Karlekar (2017) United States	*Content:* Implement validated screening process on all patients at least 65 years of age who were admitted to trauma intensive care unity (TICU) and stepdown unit Medical receptionists distributed screening forms and Trauma care providers evaluated screening results daily to determine if early palliative care referrals needed Patients who received palliative care consult were contacted 6 months after discharge to determine condition and/ or mortality status *Duration:* Duration of hospital admission *Delivered by:* Trauma nurses who were trained in screening process	Quasi‐experimental pre/post	Bedside nurses in trauma unit received group and individual training in using FRAIL questionnaire and AD8 Dementia Screen Project coordinators attended trauma and palliative care provider meetings to explain project and encourage use of screening tools to refer to palliative care services and use screening results as discussion point in palliative care discussions	Evidence shows earlier referrals to palliative care that engage patients and families in discussions around goals of care can potentially reduce 30‐day readmissions and result in significant cost saving	Number of organisations: 1 hospital, TICU and stepdown unit palliative care providers Number of interventionists: Not specified	Primary outcomes: Number of patients screened Number of palliative care consultations 54% of screened patients (35 patients) received palliative care consult compared to 14% of non‐screened (9 patients) (*p* < .001) Palliative care consultations for older adults increased from 13% before study to 33% during study
Keiser (2023) United States	*Content:* Charge nurse notified of new patient admission and would complete FRAIL questionnaire. If the patient scored 4 or higher, CN would notify primary nurse and NSCCU intensivist. Palliative care consult would then be ordered through electronic health record *Duration:* Screening conducted upon admission *Delivered By:* Charge nurse, primary nurse, and NSCCU intensivist	Pre‐/postquality improvement	All providers required to complete education program. Education included course developed by the Center to Advance Palliative Care to inform staff on palliative care services and who can benefit from referral. Deadline was given for completing the training Providers were emailed a powerpoint presentation and visual aid handouts explaining FRAIL questionnaire and new responsibilities	American Heart Association and the American Stroke Association guidelines stating that all patients with a new diagnosis of stroke should receive palliative care starting in the acute phase of care	Number of organisations: 1 hospital (14‐bed Midwestern neuroscience critical care unit in a Joint Commission Certified Comprehensive Stroke Center) Number of interventionists: 54 providers (nursing and medical staff)	Primary outcomes: In 12‐week period prior to implementation 17/114 patients admitted to NSCCU with diagnosis of acute stroke received palliative care consult orders (15%) In 12‐week period following implementation 65 out 120 patients had FRAIL assessment completed (54%). 9 patients identified as scoring 4 or higher on FRAIL assessment. 26 patients (22%) had palliative care consult: 12 (46%) for patients who had FRAIL assessments conducted and 14 (54%) for patients without an assessment. Increase in consults was not statistically significant Secondary outcomes: 55 pre and post implementation Self‐Perceived End‐of‐Life Care Competencies in the ICU surveys completed by providers. 14 providers completed both pre and post, with a significant increase in providers who agreed or strongly agreed that they were well‐prepared to treat pain in patients with stroke with nonpharmacological measures, treat gastro‐intestinal symptoms, provide grief and bereavement support to patients and families, and that continuity of care at end of life is observed when nursing assignments are made
McGrath (2019) UK	*Content:* Comprehensive geriatric assessment (CGA). Multidimensional assessment and intervention delivered by multidisciplinary team associated with improved outcomes. Embed within emergency departments Identify frailty with Clinical Frailty Scale (CFS), identified based on symptoms and function status on 9‐point scale Embed system that allowed frailty identification in older patients in ED, creation of safe clinical pathway for CGA and appropriate same‐day discharge of suitable patients, measurement to demonstrate service improvement *Duration:* Duration of ED admission *Delivered By:* Clinical staff in ED	Quasi‐experimental pre/post	Used plan‐do‐study‐act (PDSA) cycles Core project team met weekly to discuss data from previous week and problem‐solve Creation of CFS template for electronic record system and promotion of this template through multiple means Developed ‘Frailty Highlights’ report and sent out fortnightly to teams Hard copy of checklist developed Incorporated CFS prompt into patient safety checklist. Prompting by ED admin team for clinicians to complete the CFS Creating electronic case load folder ‘Medical frailty’ to distinguish patients during handover from ED to frailty pathway ‘Superweeks’ to focus on specific clinical areas, specialists leading huddles, became regular part of service, handover directly from ED consultant to geriatrician	Designed and implemented as a QI project following National Health Service Model for Improvement	Number of organisations: 1 hospital, The Whittington Hospital in Islington, North London. 15 assessment spaces in majors, 8 bed clinical decision unit within ED. 34 bed medical admissions unit or directly admitted to inpatient specialist ward Number of interventionists: Not specified. Multiple	Primary outcomes: Percentage of patients screened for frailty Attendance to admission conversion rate Increase percentage of older patients (aged over 75 years) attending ED who had CFS completed to 70% in 9 months Decrease attendance to admission conversion rate by 2% in 9 months
Street (2023) UK	*Content:* Acute Frailty Network membership supporting the delivery of evidence‐based care for older people with frailty *Duration:* N/A *Delivered by:* Experts and multidisciplinary teams	Staggered difference‐in‐difference panel event study	Adoption of the principles of the AFN. The included hospital sites needed to have adopted 4 or more of the principles of the AFN	Plan‐Do‐Study‐Act cycles and the Model for improvement	Number of organisations: 6 hospital sites Number of Interventionists: Not specified. Multiple	Primary outcomes: Length of stay, in‐hospital mortality, institutionalisation and 30‐day readmission No significant effects of AFN membership were found for any of the four outcomes, nor were there significant effects for any individual cohort
Wilson (2021) United States	*Content:* Implemented frailty assessment tool for patients 65 years and older. Identified patients with frailty received evidence‐based clinical orders and nursing care plan interventions tailored to individual's recovery Patients identified as intermediately frail or frail had dedicated order set and optional individualised orders added to their admission order set *Duration:* Duration of hospital length of stay *Delivered by:* Assessment completed by resident physicians and advanced practice providers on preoperative evaluation	Quasi‐experimental pre‐/poststudy with a process evaluation	Resident physicians and advanced practice providers trained and informational sessions conducted on frailty and assessment (ongoing throughout project) Quality improvement team convened monthly to foster and track implementation Nurses provided education through nursing huddles and formal education sessions (ongoing throughout project) Pilot phase to demonstrate uptake of assessment and accurate identification Charts of all eligible patients reviewed to determine if assessment conducted, frailty score, and frailty‐related orders that were completed	Direct orders added to order set and nursing care plan developed based on American College of Surgeons (ACS) National Surgical Quality Improvement Program (NSQIP) best practice guidelines, literature review, and consensus from surgical frailty quality improvement team	Number of organisations: 1 hospital, general and vascular surgery services Number of interventionists: Not specified. Multiple	Primary outcomes: Length of stay, favourable discharge, loss of independence, inpatient complications, 30‐day complications, readmissions, death within 30 days of discharge There was significantly decreased inpatient complications (OR 0.548, *p* = .001) and complications within 30 days of discharge (OR 1.532, *p* = .08) in the intervention group There was a non‐significant decrease in length of stay, favourable discharge, readmission within 30 days of discharge and mortality within 30 days of discharge

**TABLE 2:** Implementation strategies identified across studies using Expert Recommendations for Implementing Change (ERIC) categories.

Implementation strategies	Studies that reported using this strategy	Number of studies
1. Use evaluative and iterative strategies		
Develop and implement tools for quality monitoring	Bakker (2014), Bryant (2019), Chen (2014), Engelhardt (2018), Ernst (2014), Fritsche (2023), Hall (2017), Hall (2022), Heim (2016), Karlekar (2017), Keiser (2023), McGrath (2019), Wilson (2021)	13
Audit and provide feedback	Bakker (2014), Bryant (2019), Chen (2014), Engelhardt (2018), Hall (2022), Heim (2026), McGrath (2019), Wilson (2021)	8
Conduct local needs assessment	Bakker (2014), Bryant (2019), Chen (2014), Hall (2017), Hall (2022), Heim (2016), McGrath (2019), Street (2023)	8
Purposefully re‐examine the implementation	Bakker (2014), Engelhardt (2018), Ernst (2014), Heim (2016), McGrath (2019), Wilson (2021), Street (2023)	7
Stage implementation scale‐up	Bryant (2019), Ernst (2014), Heim (2016), McGrath (2019), Street (2023)	5
Assess for readiness and identify barriers and facilitators	Engelhardt (2018), Hall (2022), Heim (2016), Street (2023)	4
Obtain and use patients/consumers and family feedback	Bakker (2014), Harper (2023), Heim (2016), Street (2023)	4
Develop and organise quality monitoring systems	Heim (2016), McGrath (2019), Wilson (2021)	3
Develop a formal implementation blueprint	Bakker (2014), Heim (2016), McGrath (2019)	3
Conduct cyclical small tests of change	Heim (2016), McGrath (2019), Street (2023)	3
2. Provide interactive assistance		
Facilitation	Bakker (2014), Bryant (2019), Engelhardt (2018), Hall (2022), Heim (2016), McGrath (2019), Wilson (2021), Street (2023)	8
Provide clinical supervision	Bakker (2014), Bryant (2019), Chen (2014), Heim (2016), Street (2023)	5
Provide local technical assistance	Heim (2016), Street (2023)	2
Centralise technical assistance	Street (2023)	1
3. Adapt and tailor content		
Promote adaptability	Bakker (2014), Engelhardt (2018), Ernst (2014), Hall (2017), Hall (2022), Heim (2016), Karlekar (2017), Wilson (2021), Street (2023)	9
Tailor strategies	Bryant (2019), Engelhardt (2018), Hall (2022), Heim (2016), McGrath (2019), Wilson (2021), Street (2023)	7
Use data experts	Hall (2022), Street (2023)	2
4. Develop stakeholder interrelationships		
Organise clinician implementation team meetings	Bakker (2014), Bryant (2019), Engelhardt (2018), Hall (2022), Heim (2016), Karlekar (2017), McGrath (2019), Wilson (2021)	8
Capture and share local knowledge	Bakker (2014), Cooper (2021), Engelhardt (2018), Ernst (2014), Hall (2022), Heim (2016), Street (2023)	7
Use advisory boards and workgroups	Bakker (2014), Bryant (2019), Engelhardt (2018), Fritsche (2023), Heim (2016), McGrath (2019), Wilson (2021)	7
Conduct local consensus discussions	Engelhardt (2018), Fritsche (2023), Hall (2017), Heim (2016), McGrath (2019), Wilson (2021)	6
Identify and prepare champions	Bakker (2014), Bryant (2019), Engelhardt (2018), Heim (2016), McGrath (2019)	5
Recruit, designate, and train for leadership	Bryant (2019), Ernst (2014), Hall (2017), Heim (2016), McGrath (2019)	5
Promote network weaving	Fritsche (2023), Hall (2022), Heim (2016), Street (2023)	4
Use an implementation advisor	Hall (2022), McGrath (2019), Street (2023)	3
Build a coalition	Heim (2016), Street (2023)	2
Inform local opinion leaders	Street (2023)	1
Obtain formal commitments	Fritsche (2023)	1
Visit other sites	Heim (2016)	1
Involve executive boards	Street (2023)	1
Develop academic partnerships	Heim (2016)	1
Identify early adopters		0
Model and simulate change		0
Develop an implementation glossary		0
5. Train and educate stakeholders		
Develop educational materials	Bryant (2019), Ehrlich (2023), Engelhardt (2018), Ernst (2014), Fritsche (2023), Hall (2022), Heim (2016), Keiser (2023), McGrath (2019)	9
Distribute educational materials	Bryant (2019), Ehrlich (2023), Engelhardt (2018), Ernst (2014), Fritsche (2023), Hall (2022), Keiser (2023), McGrath (2019), Street (2023)	9
Conduct ongoing training	Bakker (2014), Bryant (2019), Chen (2014), Hall (2022), Heim (2016), McGrath (2019), Wilson (2021), Street (2023)	8
Provide ongoing consultation	Bakker (2014), Bryant (2019), Chen (2014), Hall (2022), Heim (2016), McGrath (2019), Wilson (2021), Street (2023)	8
Conduct educational meetings	Bakker (2014), Fritsche (2023), Hall (2022), Heim (2016), Karlekar (2017), McGrath (2019), Wilson (2021), Street (2023)	8
Conduct educational outreach visits	Bryant (2019), Fritsche (2023), Hall (2022), Heim (2016), McGrath (2019), Wilson (2021), Street (2023)	7
Make training dynamic	Bryant (2019), Chen (2014), Fritsche (2023), Hall (2022), Heim (2016), McGrath (2019)	6
Create a learning collaborative	Hall (2022), Heim (2016), Street (2023)	3
Use train‐the‐trainer strategies	Street (2023)	1
Work with educational institutions	Heim (2016)	1
Shadow other experts		0
6. Support clinicians		
Revise professional roles	Ernst (2014), Hall (2017), Heim (2016), Karlekar (2017), Keiser (2023), McGrath (2019)	6
Create new clinical teams	Bakker (2014), Chen (2014), Heim (2016), McGrath (2019), Street (2023)	5
Facilitate relay of clinical data to providers	Engelhardt (2018), Heim (2016)	2
Remind clinicians	Karlekar (2017), McGrath (2019)	2
Develop resource‐sharing agreements	Fritsche (2023), Heim (2016)	2
7. Engage consumers		
Involve patients/consumers and family members	Street (2023)	1
Intervene with patients/consumers to enhance uptake and adherence		0
Prepare patients/consumers to be active participants		0
Increase demand		0
Use mass media		0
8. Utilise financial strategies		
Access new funding	Bakker (2014), Chen (2014), Ehrlich (2023), Engelhardt (2018), Heim (2016), Street (2023)	6
Fund and contract for the clinical innovation	Street (2023)	1
Place innovation on fee for service lists/formularies		0
Alter incentive/allowance structures		0
Make billing easier		0
Alter patient/consumer fees		0
Use other payment schemes		0
Develop disincentives		0
9. Change infrastructure		
Mandate change	Bakker (2014), Bryant (2019), Chen (2014), Ernst (2014), Fritsche (2023), Hall (2017), Heim (2016), McGrath (2019), Wilson (2021), Street (2023)	10
Change record systems	Heim (2016), McGrath (2019), Wilson (2021)	3
Change physical structure and equipment	Heim (2016)	1
Change service sites	Heim (2016)	1
Change accreditation or membership requirements	Street (2023)	1
Start a dissemination organisation	Street (2023)	1
Create or change credentialling and/or licensure standards		0
Change liability laws		0

**TABLE 3:** Results of the implementation outcomes in RE‐AIM dimensions.

Implementation Study	Did the study use RE‐AIM?	Reach (number/proportion of patients willing to receive the intervention)	Effectiveness (outcomes)	Adoption (number/proportion of organisations and interventionists willing to initiate the intervention)	Implementation (fidelity to the intervention)	Maintenance
Bakker (2014) The Netherlands	No	Preimplementation group: 370 patients screened, 298 met eligibility criteria (81%) and 191 (64%) were included in the analysis. Total of 27 patients refused to participate and 3 dropped‐out Postimplementation group: 430 admissions, 303 (71%) met eligibility criteria (71%) and 195 (64%) were included in the analysis. Total of 65 patients refused to participate and 12 dropped‐out Reasons for refusal to participate not given	There was no statistically significant decrease in primary effect outcomes of: (1) incidence of hospital‐acquired delirium (*p* = .945), (2) incidence of cognitive decline (*p* = .43) (3) or incidence of ADL decline (*p* = .09) In secondary effect outcomes there was improvement of function at 3 months (*p* = .06) and reduction in burden of care at 3 months (*p* = .49). There was no significant change in readmission rate (*p* = .43)	Number of organisations: 1 Implementation in 3 departments within a single hospital Number of Interventionists: A CareWell team consisting of 2 members (a geriatric nurse specialist and a geriatrician) Trained volunteers Number approached who were eligible to be trained as interventionalists: Not reported	Adherence to the CWH ranged from 11% (Multidisciplinary meeting) to 95% (Development of the CareWell plan) Adherence to the indicated intervention by departments: Range 37%–100% Dietetics 100% (8/8 referred) Dietitian consult 37–40% (7/19) Screening by nurses and adherence to recommendations increased over time: From 61% in September 2011 to 76% in May 2012 Integrating the work of volunteers: 46% adherence rate	Screening percentages increased in the postimplementation phase The program was expanded into two additional departments following conclusion of the trial
Bryant (2019) United States	No	125 patients were included in the preintervention cohort (there were 242 patients aged 65 or older. Of these 150 received geriatric assessment and 125 were with prefrailty or frailty) 144 patients were included in the postintervention cohort (there were 218 patients aged 65 or older. Of these, 204 were screened for frailty, 190 screened positive, 161 admitted to trauma unit and evaluated by geriatrician. 6 patients excluded as they transferred to other services or deemed robust after geriatric assessment)	In univariate analysis, there were no significant differences in complications (28% vs. 29%, respectively, *p* = .93); however, there was a significant decrease in delirium (22%–13%, respectively, *p* = .04) and 30‐day readmission (10%–3%, respectively, *p* = .01)	Number of Organisations: 1 Number of Interventionists: Multiple, including a nurse champion and trauma geriatrician Number approached who were eligible to be trained as interventionalists: Not specified	During implementation, 204/218 (93.6%) patients aged 65 years or older were screened for frailty. Adherence to other components of care pathway is not specified	Not specified
Chen (2014) Taiwan	No	217 patients were eligible. 28 declined participation in the study. 189 participants were randomised. 82 received usual care, 107 receive the intervention In the usual care group, there were 3 deaths and 3 drop‐outs during hospital admission, and 4 deaths and 0 drop‐outs in the 3 months following discharge In the intervention group there were 4 deaths and 1 drop‐outs during hospital admission, and 7 deaths and 3 drop‐outs in the 3 months following discharge	There was a significant reduction in frailty and transitions to higher frailty states on discharge in the intervention group, as compared to the control group For matched pairs, participants who received the mHELP interventions were significantly less likely to be with frailty at discharge (19%) than matched controls (65%) (*p* < .001) There was no statistically significant difference in frailty state between the intervention and control group at 3 months after discharge	Number of organisations: 1 Number of Interventionists: 1 Number approached who were eligible to be trained as interventionalists: Not specified	During the intervention there was one drop‐out The majority of participants (54%) received approximately 7 days of the mHELP program (range 4–20)	Frailty status was measured 3 months following intervention and there was no significant difference between control and intervention group The intervention was not continued after the intervention period
Ehrlich (2023) United States	No	300 patients retrospectively screened for frailty through electronic health records for pre pathway cohort. 233 patients screened once cohort implemented Patients who were not screened were not included but did not report how many patients were not screened 80 patients pre pathway implementation had frailty (27%) and 74 patients had frailty post pathway implementation (32% of patients screened)	Patients with frailty who were part of GSP cohort had significantly decreased risk for loss of independence, major complications, and reduced length of stay. There was no significant different in readmission rates	Number of organisations: 1 (Implemented across several departments within one hospital) Number of interventionists: Multiple interventionists from different disciplines. Numbers not specified Number approached who were eligible to be trained as interventionalists: Not specified	Not specified	Not specified
Engelhardt (2018) United States	No	100% patients ≥65 years, screened for frailty for pre and post period of 3 months in geriatric trauma and emergency general surgery 239 patients screened, 70 (29%) identified as with frailty (pre and post intervention) During implementation, all patients with frailty are to use frailty pathway	Following implementation of frailty pathway, patients with frailty (*n* = 59) had decreased median length of stay from 9 to 6 days (*p* = .4), decreased readmissions from 36% to 10% (*p* = .02), and loss of independence decreased by 40% (*p* = .01) Outcomes did not change for patients without frailty	Number of Organisations: 1 hospital Number of Interventionists: multiple staff involved, specific physician champion who ensured reaching 100% screening rate Number approached who were eligible to be trained as interventionalists: Not specified	Compliance during implementation to components of the frailty pathway ranged from 100% with screening, 100% with hospitalist consults, and 97% with expectation‐setting conversations. 9% of patients had a palliative care consult but this was not a compulsory step in frailty pathway. Did not measure compliance for specialised order set or postdischarge follow‐ups	Focusing on sustaining implementation of the frailty pathway but no data available
Ernst (2014) United States	No	Screened initially with patients with hip fracture, as of 1 July 2011 screened all patients presenting to surgical evaluation unit for elective surgery. This method captured 65% of elective operations. As of 1 February 2012, also screened patients presenting to individual surgical clinics, capturing frailty scores for 90% of elective surgery patients. As of 1 July 2012, frailty scores required for scheduled surgical procedures, capturing all but emergency procedures Chief of surgery or designee reviewed medical record for each patient with frailty to confirm surgical planning and examine potential system interventions From January 2006 to August 2013: 310 surgical palliative care consults (160 pre implementation, 15 post). Annual consult rate of 32 pre implementation and 56 post (significantly more)	Approximately 10% of patients were identified as with frailty From January 2006 to August 2013: 310 surgical palliative care consults (160 pre implementation, 15 post). Annual consult rate of 32 pre implementation and 56 post (significantly more) Significant decrease in mortality rate amongst patients with palliative care consult at 30 days (32% pre and 21% post), 180 days (71% pre and 44% post), and 360 days (79% pre and 66% post). Significantly more patients did not have surgery post implementation (6% pre and 19% post) Mean survival days not significantly different (295 pre and 314 post) Implementation of the screening program was associated with a significantly reduced odds of death Preoperative palliative care consultations ordered by a surgeon were associated with the greatest reduction in mortality (OR, 0.27; 95% CI, 0.11–0.68; *p* = .006)	Number of organisations: 1 hospital: Nebraska Western Iowa Veterans Affairs Medical Centre Number of interventionists: Not specified Number approached who were eligible to be trained as interventionalists: Not specified	Screened initially with patients with hip fracture, as of 1 July 2011 screened all patients presenting to surgical evaluation unit for elective surgery. This method captured 65% of elective operations. As of 1 February 2012, also screened patients presenting to individual surgical clinics, capturing frailty scores for 90% of elective surgery patients. As of 1 July 2012, frailty scores required for scheduled surgical procedures, capturing all but emergency procedures Chief of surgery or designee reviewed medical record for each patient with frailty to confirm surgical planning and examine potential system interventions From January 2006 to August 2013: 310 surgical palliative care consults (160 pre implementation, 150 post). Annual consult rate of 32 pre implementation and 56 post (significantly more) Following implementation, palliative care consults more frequently ordered by surgeons (24% pre and 57% post)	Implies project is ongoing, but no follow‐up data available
Fritsche (2023) The Netherlands	No	From January to March 2021 (preimplementation) 1358 patients with frailty were admitted and from October to December 2021 (postimplementation) 1417 patients with frailty were admitted. A computerised random sample of 200 patients from each time period were included in analysis (14% of eligible patients) Communication between health‐care providers was retrospectively checked for the 400 patients with frailty seen before and after implementation of the agreement between services	Before implementation of the agreement, frailty was mentioned in 13% of letters from the hospital compared to 15% of letters postimplementation (not significant) There was no significant difference in mentions of medication lists or resuscitation orders before and after implementation Number of referral letters from GP improved significantly (32%–54%). Mentions of frailty in referral letters was not significantly different before and after implementation No significant differences in discharge letters being sent within 24 h before and after implementation (57%–61%)	Number of organisations: 1 hospital, all departments Number of interventionists: Not specified Number approached who were eligible to be trained as interventionalists: Not specified	Following implementation, only 15% of letters to GPs for patients with frailty mentioned patients' frailty There were no improvements in mentions of medication lists or resuscitation orders in letters nor in timely discharge letters being sent There was a significant improvement in referral letters from GPs received (increase from 32% to 54%)	State that following adjustments to improve the implementation, the protocol will be re‐implemented and if it is successful, impacts on health outcomes can be evaluated
Hall (2017) United States	No	9153 patients presented for major elective non‐cardiac surgery from 1 October 2007 to 1 July 2014 5275 patients before screening was implemented‐ measured frailty retrospectively by mapping Veterans Affairs Surgical Quality Improvement Program variables to each of 14 items on RAI and calculating RAI score All patients who attended once screening implemented (from July 2011) were assessed for frailty	Modelling of data showed increasing frailty associated with increasing risk of death. Risks were significantly reduced after frailty screening implemented 30‐day mortality significantly reduced from 2% (84 of 5275 patients before screening) to 1% (26 of 3878 patients with screening). Improvement greatest among patients with frailty: 12% (24/197) to 4% (16/424) (mortality also decreased for patients without frailty). Improvement in mortality rate increased for patients with frailty at 180 days (24% prescreening to 8% post) and at 365 days (35%–12%)	Number of Organisations: One hospital. Veterans Affairs Nebraska‐Western Iowa Health Care System in Omaha, Nebraska Number of Interventionists: Not specified Number approached who were eligible to be trained as interventionalists: Not specified	Beginning in July 2011, 100% of patients who presented for elective non‐cardiac surgery were screened for frailty	Not specified
Hall (2022) UK	No	Between 2018 and 2021, 50 clinical teams undertook quality improvement projects for frailty with the support of the Specialised Clinical frailty Network (SCFN) Not specified how many patients were seen or identified through new pathways	All 10 frailty principles developed by the SCFN saw improvements in percentage of teams that were adhering to principles (e.g. 28% of teams had mechanisms for identifying people with frailty pre‐SCFN compared to 95% of teams post) Some teams saw clinical outcome improvements from implementation (e.g., length of stay decreased from 3 to 2 days in Barts Health NHS Trust transcatheter aortic valve implantation (TAVI) service)	Number of organisations: Not specified Number of Interventionists: 50 clinical teams Number approached who were eligible to be trained as interventionalists: Not specified	Following implementation, alignment with the 10 SCFN principles ranged from 56% to 95% across the teams	Not specified
Heim (2016) The Netherlands	No	2010–2011: 721 completed 3‐month follow‐up questionnaire with complete functional decline data, health‐care demand after admission available for 713 patients, data on mortality available for 816 2012–2013: 827 completed 3‐month follow‐up questionnaire with complete functional decline data, health‐care demand after admission available for 785 patients, data on mortality available for 916 813 (2010–11) and 904 (2012–13) patients included in analyses of adverse outcomes after 3 months. Patients who were only known to be alive were excluded from analyses	Prevalence of frailty 38% (2010–11) and 42% (2012–13) Incidence of adverse outcomes for patients with frailty was 49% (149/303) preprogram and 36% (130/366) postprogram Reduced risk for patients with frailty across all hospitals and all outcomes (decline of ADL, mortality, high health‐care demand) Use of professional care more prevalent in postprogram sample in first 58 days after admission, rest of period no difference Average costs lower for postprogram sample for first 6 months, but higher after 6 months Overall: no significant difference in average costs	Number of Organisations: 4 hospitals and other health‐care providers Number of Interventionists: Not specified Number approached who were eligible to be trained as interventionalists: Not specified	As there were a wide number of innovations implemented across sites, there is no record of how many patients had access / were referred through each specific innovation or pathway	Encouraged to permanently embed new innovations in health‐care organisations. No details on follow up
Karlekar (2017) UK	No	From March to May 2015: 131 admissions of older people 49% of older people were screened (64 patients)	From March to May 2015: 49% of 131 older people were screened for frailty 38% screened positive for frailty, 45% screened positive for possible dementia, 23% screened positive for both conditions 54% of screened patients (35 patients) received palliative care consult compared to 14% of non‐screened (9 patients) (*p* < .001) Palliative care consultations for older adults increased from 13% before study to 33% during study 6‐month phone‐call follow up, 16% lost to follow up. Of remaining 37, 17 (46%) passed away by 6 months	Number of Organisations: 1 hospital Number of Interventionists: Not specified, no details on uptake by clinical staff to use screening tools Number approached who were eligible to be trained as interventionalists: Not specified	From March to May 2015: 49% of 131 older people were screened for frailty 18/25 patients with frailty had palliative care consult, 11/21 with prefrailty, 18/24 with possible dementia	No follow up details
Keiser (2023) United States	No	In 12‐week period following implementation 120 patients were admitted to the unit with a diagnosis of acute stroke, 65 patients (54%) were screened	In 12‐week period prior to implementation 17/114 patients admitted to NSCCU with diagnosis of acute stroke received palliative care consult orders (15%) In 12‐week period following implementation 65 out 120 patients had FRAIL assessment completed (54%). 9 patients identified as scoring 4 or higher on FRAIL assessment. 26 patients (22%) had palliative care consult: 12 (46%) for patients who had FRAIL assessments conducted and 14 (54%) for patients without an assessment. Increase in consults was not statistically significant 55 pre and post implementation Self‐Perceived End‐of‐Life Care Competencies in the ICU surveys completed by providers. 14 providers completed both pre and post, with a significant increase in providers who agreed or strongly agreed that they were well‐prepared to treat pain in patients with stroke with non‐pharmacological measures, treat gastro‐intestinal symptoms, provide grief and bereavement support to patients and families, and that continuity of care at end of life is observed when nursing assignments are made	Number of Organisations: 1. Neuroscience Critical Care Unit Number of Interventionists: 54 providers Number approached who were eligible to be trained as interventionalists: All providers on unit were required to complete education program	FRAIL assessment was completed for 65/120 (54.2%) of patients over 12‐week period from implementation 9 patients identified as scoring 4 or higher on FRAIL assessment. 26 patients (22%) had palliative care consult: 12 (46%) for patients who had FRAIL assessments conducted and 14 (54%) for patients without an assessment	Not specified
McGrath (2019) United States	No	By Jan 2019: 319 patients identified and transferred 1653 patients out of eligible 3577 patients had CFS completed	Admission rate for patients on frailty pathway around 14% (compared to overall admission rate of 50%) 53 patients discharged on same day through pathway had a 7‐day readmission rate of 15% and 30‐day rate of 19%. This readmission rate is higher than for all ED patients over 75 years but lower than discharges from inpatient wards Notes review estimated 46/53 patients avoided longer admission due to pathway Overall, ED conversion rate reduced from 51% to 49% (decrease of overall ED admission rate by 1%) From 10 patients: 90% happy with experience of frailty pathway, 80% felt time to complete CGA was acceptable, 100% described experience as better or same as previous ED attendance From 22 staff: 77% felt confident using the CFS, 100% felt frailty team was beneficial to patient flow	Number of Organisations: 1. ED department of Whittington hospital, North London Number of Interventionists: Not specified Number approached who were eligible to be trained as interventionalists: Not specified	Percentage of patients with CFS completed greatest in Superweek months when more emphasis placed on completing CFS and when admin team reminded and huddles commenced. Lower fidelity in December when pathway not well staffed and more staff on leave Percentage of patients with CFS completed ranged from approx. 20%–75% across different months	Implies program is ongoing
Street (2023) UK	No	6 hospital sites were the 6 implementation cohorts. Two sites were control sites Total patients 1,410,427 968,900 patients were ‘never treated’ controls, 336,236 were ‘not‐yet‐treated’ controls and 10,292 were in the intervention group	No significant effects of AFN membership were found for any of the four primary outcomes: Length of stay, in‐hospital mortality, institutionalisation, and 30‐day readmission There were no significant effects for any individual cohort	Number of Organisations: 6 cohorts of hospitals. Number of individual hospitals not specified Number of Interventionists: Not specified. Multiple Number approached who were eligible to be trained as interventionalists: Not specified	Self‐report data about adoption and implementation at participating hospital sites. No objective observation or measurement of fidelity	Program is ongoing
Wilson (2021) United States	Yes	Preimplementation group: 316 would have been eligible Postimplementation group: 1158 patients met inclusion criteria. 696 (60%) had assessment completed	In postintervention period there were significant increases in rate of delirium prevention orders, orders to avoid overnight vital signs, aspiration precaution orders, nutrition consults, social work consults, and pharmacy consults Postintervention saw patients with frailty receive higher rates of frailty specific orders than robust and non‐assessed patients Postintervention significantly associated with decreased inpatient complications and complications within 30 days of discharge, significantly decreased rates of acute respiratory failure/ mechanical ventilation and sepsis within 30 days of discharge No significant pre‐/postdifference in length of stay, favourable discharge, loss of independence, readmissions within 30 days, and death within 30 days of discharge Surveys indicated high rates of awareness (100%, *n* = 28) and acceptability of frailty assessment	Number of organisations: 1 hospital: two services (general and vascular surgery) within this hospital Number of Interventionists: 11 stakeholders developed and trained all eligible resident physicians and advanced practice providers. All nurses were educated by nurse education or research team. Total number not specified Number approached who were eligible to be trained as interventionalists: Not specified	Delirium assessments document in 32% of patients with order for delirium assessment Frailty nursing care plan implemented for 32% of patients with order Patients with no overnight vitals, 99% still had vital signs documented overnight 91% adherence to aspiration precautions Patient orientation 83% adherence	Follow‐up data not available

The Standards for Reporting Implementation Studies (STaRI)^15^ emphasises the importance of examining both strands of interest in implementation studies: the implementation strategy and process, as well as the clinical intervention being implemented. Through extracting data in Tables 1, 2, 3, both the clinical interventions and implementation information are explored for the included studies.

### 2.4 Assessment of risk of bias and reporting

The authors appraised the risk of bias using the National Institute of Health's (NIH) Quality Assessment Tool for Before–After (Pre/Post) Studies with No Control Group for all studies evaluating outcomes before and after implementing frailty interventions. However, one study used a non‐randomly selected control group and was appraised for risk of bias using the NIH Quality Assessment of Controlled Intervention Studies (no RCTs were included in this review).^21^ These tools were adapted, and we only reported on items relevant to the internal validity of each study, as has been done in previous systematic reviews.^22^, ^23^ The NIH Quality Assessment Tool is recommended for assessment of bias in Pre‐/Poststudies without control groups.^24^ Both tools are available in Data S3. Risk of bias was assessed by one reviewer.

### 2.5 Data synthesis

A narrative synthesis approach was used due to the heterogeneity of the included studies, particularly in relation to interventions, implementation strategies, methods and outcomes. The characteristics of individual studies were tabulated, and the ERIC and RE‐AIM frameworks were applied to structure the identification of relevant information and the recognition of patterns across studies.

## 3 RESULTS

### 3.1 Search results and study selection

**FIGURE 1:**
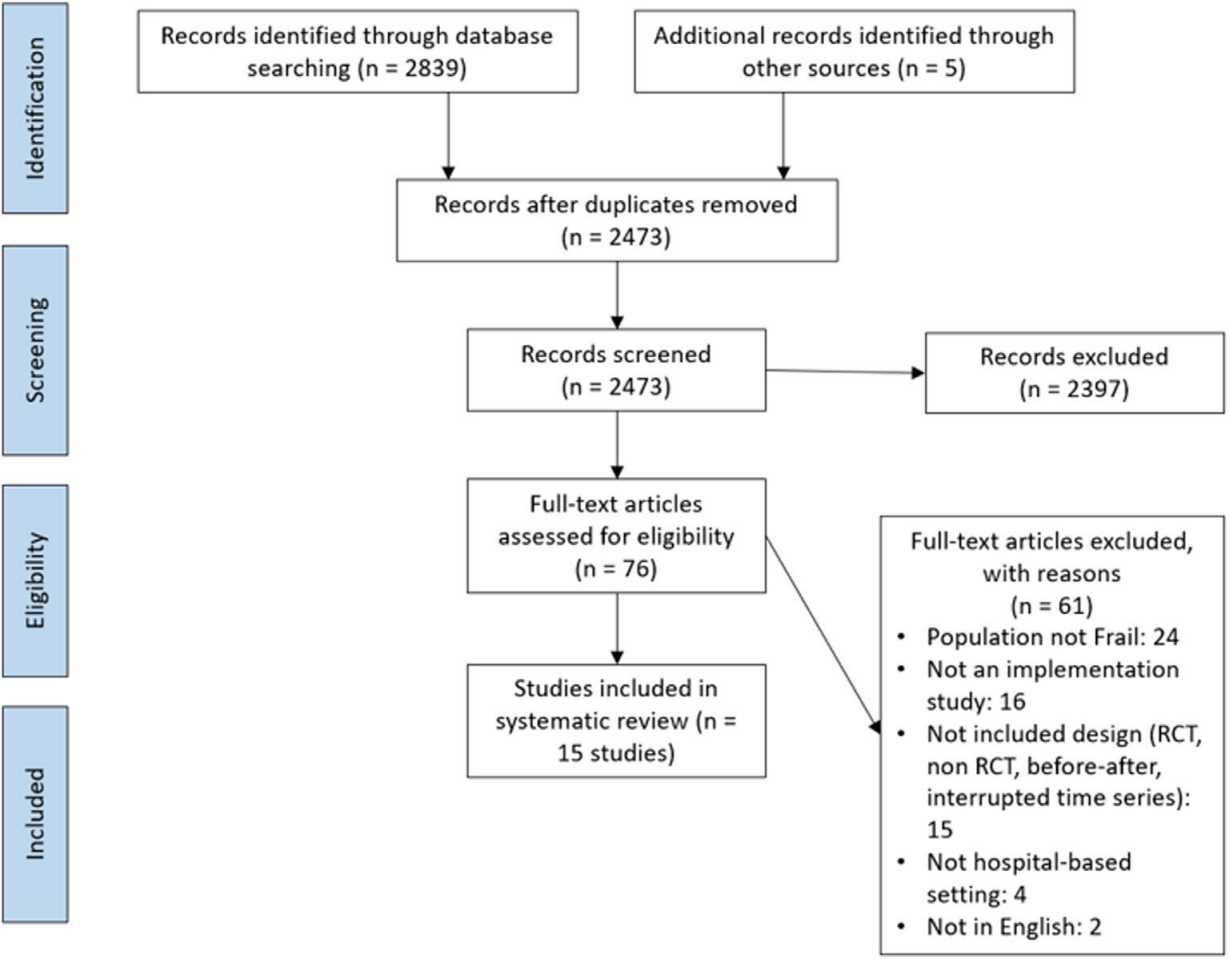
PRISMA flow diagram.

Database searching retrieved 2839 articles. After removing duplicates using the Covidence software, 2473 titles and abstracts were screened for eligibility. We identified 76 articles for full‐text review and, of these, 15 articles met the requirements for inclusion in the review. Reference lists and grey literature were searched, and one further article was identified and reviewed; however, it did not meet the requirements for inclusion in the review. See Figure 1 for the PRISMA flow diagram.

### 3.2 Setting

Eight studies were carried out in the United States,^25^, ^26^, ^27^, ^28^, ^29^, ^30^, ^31^, ^32^ three studies in the Netherlands,^33^, ^34^, ^35^ three in the UK^36^, ^37^, ^38^ and one in Taiwan.^39^ All studies were published in the last decade (2014 onward).

### 3.3 Study design

All included studies were quasi‐experimental pre/post or quality improvement studies. Fourteen studies used preintervention and postintervention measurements and one used a non‐randomly selected control group.^27^

All of the studies reported on data preintervention and postintervention, some with longer follow‐up periods for specific outcomes, for example 30‐day readmission^28^, ^29^, ^30^, ^31^, ^38^ and 30‐day mortality.^25^, ^26^, ^28^, ^31^, ^34^ The longest follow‐up period was 365 days for mortality.^25^, ^26^

### 3.4 Risk of bias and reporting

Risk‐of‐bias assessments can be found in Data S3. All of the studies were quasi‐experimental or quality improvement designs, with lack of random assignment being a limitation of these studies.^40^ In quasi‐experimental studies, there may be alternative explanations to apparent causal associations as these trials are often unable to control for potential confounding variables.

We were able to identify information relating to the internal validity of each study using items from the NIH Quality Assessment Tools for Before–After Studies with No Control Group and Controlled Intervention Studies.^21^ Information on the rigour of each study's methodology was consistently poorly described. Most studies addressed study design as a limitation in their discussion section.^25^, ^26^, ^28^, ^30^, ^31^, ^33^, ^34^, ^35^, ^38^, ^39^

Each study reported clear eligibility criteria, and clinical outcomes were measured before and after the prescribed intervention. Clinical outcome measures were usually prespecified, clearly defined and consistently assessed across all study participants. Overall, studies did not report study sample size justifications or power calculations, and there was no (or unclear) blinding of outcome assessors, as well as high loss to follow‐up (Data S3). We considered the included studies to be low‐quality evidence.

### 3.5 Clinical interventions

Most studies reported a lack of routine identification of patients with frailty in their hospital settings and then attempted to address this deficit by implementing a frailty screening system as part of their intervention. There were a variety of tools used to screen for frailty: the FRAIL scale,^27^, ^29^, ^32^ a frailty assessment tool adapted from the FRAIL scale,^28^ the Clinical Frailty Scale,^35^, ^36^, ^37^, ^38^ the RAI (Risk Analysis Index),^25^, ^26^ the Trauma‐Specific Frailty Index,^30^ Fried's criteria,^39^ the Edmonton Frail Scale,^31^ the Dutch Safety Management System (DSMS‐tool)^34^ and the Katz‐index and VMS+ tool in conjunction.^33^

All studies provided a frailty intervention after identifying a patient with frailty. Nine were multidisciplinary interventions aimed at addressing frailty,^28^, ^29^, ^30^, ^31^, ^33^, ^34^, ^36^, ^37^, ^38^ three studies used frailty identification as a trigger for timely palliative care consultations,^25^, ^27^, ^32^ one study implemented a nursing intervention to address frailty,^39^ another involved guidelines for documentation and information transfer about frailty during admission and on discharge,^35^ and one was a surgical preoperative review to aid surgical decision‐making.^26^

### 3.6 Nature of implementation strategies

**FIGURE 2:**
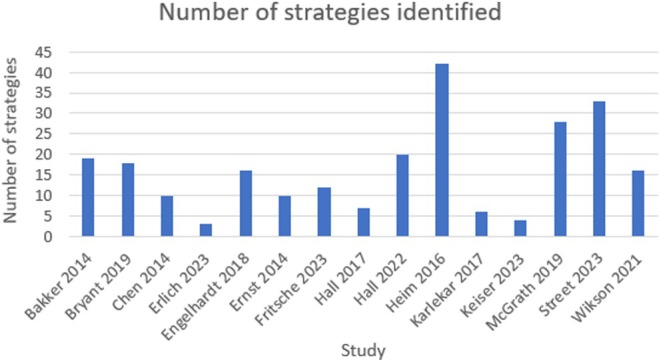
Number of ERIC strategies identified in each study.

We categorised the nature or type of implementation strategy using the ERIC categories.^19^, ^41^ Expert Recommendations for Implementing Change comprises 73 discrete strategies in nine thematic clusters and is the most commonly used taxonomy of strategies used in implementation science.^42^ As seen in Table 2, all studies used multiple implementation strategies. The number of strategies implemented ranged from 3 to 42 in a single study (Figure 2). The most common implementation strategy was *developing and implementing tools for quality monitoring*, which was used in 13 studies. Other common implementation strategies included *mandating change* (10 studies), *promoting adaptability* (9 studies) and *distributing educational materials* (9 studies).

There were 16 ERIC implementation strategies that were not identified in any of the included studies. These strategies were primarily within the categories of e*ngaging consumers* and *utilising financial strategies*.

### 3.7 Selection of implementation strategies

Most studies did not identify a framework for analysing implementation outcomes. Only one study used the RE‐AIM framework.^28^ We extracted data from all included studies and mapped this information against the RE‐AIM framework (Table 3).

### 3.8 RE‐AIM: reach

Fourteen of the studies clearly identified the population, specifying the inclusion/exclusion criteria and sample size. The participation rate was reported in 12 of the included studies.^25^, ^26^, ^28^, ^29^, ^30^, ^31^, ^32^, ^33^, ^34^, ^35^, ^38^, ^39^ Characteristics of participants were consistently outlined, but characteristics of non‐participation were less consistently addressed. Reasons for exclusion of participants were usually outlined (12 studies); however, reasons for refusal of participation were less commonly reported, with 10 studies not including this information.^23^, ^27^, ^31^, ^32^, ^33^, ^34^, ^35^, ^37^, ^38^, ^39^

### 3.9 RE‐AIM: effectiveness

The clinical effect of the interventions was reported in all studies. Fourteen of the included studies reported positive primary and/or secondary outcomes. There were a variety of outcomes assessed in these studies. Six studies examined the implementation of screening tools and identification of patients with frailty, all of them reporting increased screening and diagnosis of frailty.^25^, ^26^, ^28^, ^29^, ^30^, ^34^ Seven studies reported on emergency department (ED) reattendance and/or readmission rates following discharge from hospital,^28^, ^29^, ^30^, ^31^, ^34^, ^36^, ^38^ with three studies finding a significant reduction in readmission rate^29^, ^30^ and ED reattendance.^36^ Four studies reported on functional improvements for participants, with three studies having favourable outcomes^30^, ^31^, ^34^ and one finding no significant difference.^28^ Four studies reported on mortality, with two studies reporting a reduction in mortality rate^25^, ^26^ and two finding no difference.^28^, ^38^ Rate of complications and patient outcomes were also common outcome measures, with three studies reporting favourable outcomes^28^, ^31^, ^33^ and one reporting no difference.^29^ Three studies reported on length of stay, with two studies finding a reduction in hospital length of stay^30^, ^31^ and one finding no significant difference.^38^ Three studies that implemented frailty screening to pathways to palliative care consultations reported increases in these consults.^25^, ^27^, ^32^

Other less commonly reported outcomes included positive outcomes for reduction in frailty state on discharge^39^; however, this was not maintained at 3 months, and reduction in carer burden.^34^ One study reported an increased number of GP referral letters; however, there was no increase in mention of frailty or resuscitation status on discharge letters.^35^ Another study showed no difference in institutionalisation (discharge to hospice or care home).^38^ One study focussed on process outcomes and found positive process changes in all 10 principles assessed.^37^ No studies used quality‐of‐life outcome measures.

The length of follow‐up varied in the included studies. Eight studies had follow‐up ranging from 3 months^33^, ^34^, ^39^ to 365 days.^26^ Four studies had only 30‐day follow‐up to track re‐admission, complication and/or mortality rates.^28^, ^29^, ^30^, ^31^, ^36^ Two did not follow up after the end of the intervention period.^32^, ^35^ One study had an unclear length of follow‐up.^37^

### 3.10 RE‐AIM: adoption

Adoption can be considered at the level of the organisation and at the level of the individual health‐care provider.

In this review, the majority of studies (12 studies) reported on the implementation of an intervention within a single hospital. Three studies included multiple hospitals.^33^, ^37^, ^38^ Most studies reported adoption or change in practice in terms of the organisation level.^25^, ^26^, ^27^, ^28^, ^30^, ^32^, ^34^, ^35^, ^36^, ^37^, ^38^ The number or proportion of organisations or interventionalists willing to adopt the intervention is presented in Table 3.

All studies included a description of the health‐care providers involved in implementing the intervention. There was, however, a variable level of detail given about the level of experience or training required of the staff delivering the intervention. There was usually little information regarding methods to identify and recruit staff to deliver the intervention, inclusion/exclusion criteria for staff, and adoption rate for these roles.

### 3.11 RE‐AIM: implementation

All studies reported the intervention type and details relating to the delivery of the intervention. Most studies, however, did not report on the individual agents' fidelity to the various elements of the intervention protocol,^25^, ^26^, ^27^, ^29^, ^31^, ^32^, ^35^, ^36^, ^37^, ^39^ so it is unclear how consistently the intervention was delivered. Adaptations made to the protocol were not specifically discussed in any of the included studies. One study, however, included discussion about refinements to strategies they made throughout the process of implementation,^33^ and others referred to the quality improvement process, indicating that changes may have been made but without specific details given.

Most studies did not report on measures of cost to start up or deliver the intervention and only two studies discussed the measured cost of the intervention.^28^, ^33^

### 3.12 RE‐AIM: maintenance

Most studies had short follow‐up periods and did not examine the extent to which the interventions were maintained over time. It is therefore unknown what aspects of the interventions may have had ongoing system‐level impacts. Five of the included studies reported, or implied, that their settings are still delivering the interventions,^25^, ^30^, ^34^, ^36^, ^38^ with one of these reporting expanding their intervention since the end of their study period.^34^, ^37^ No studies reported on the cost of maintenance.

## 4 DISCUSSION

This systematic review of 15 studies examined implementation strategies for improving the care of people with frailty in hospitals. The RE‐AIM framework (Reach, Effectiveness, Adoption, Implementation and Maintenance) guided the analysis of implementation strategies and outcomes. It allowed for a structured assessment of both the clinical effectiveness of frailty interventions and important aspects of their real‐world application. Key findings from this review are summarised in Box 1 and include that most studies reported positive outcomes from frailty interventions, which invariably involved screening followed by targeted interventions. Despite the use of varied screening tools, reflecting an ongoing lack of consensus in the field,^43^ it is encouraging that studies consistently demonstrated increased frailty screening and identification rates. Multidisciplinary interventions were the most common type of intervention, which aligns with frailty guideline recommendations.^5^, ^44^ However, the predominantly quasi‐experimental designs (pre‐/poststudies or quality improvement) used in these studies limit the reliability of the findings.

BOX 1 **Key findings**

Frailty interventions in hospital settings are feasible, with most translational studies reporting positive outcomes.

Multidisciplinary interventions are the most common type of frailty intervention implemented.

Quasi‐experimental designs, including pre‐/poststudies and quality improvement projects, are the predominant study designs used.

Common frailty intervention implementation strategies include staff education, mandated practice changes, the introduction of new recording or documentation systems and monitoring practices.

Studies rarely incorporate consumer input or collect patient‐reported outcome measures.

Poor reporting quality, including limited use of established reporting guidelines and implementation frameworks, hinders the replicability and broader translation of successful frailty interventions to other settings.

The common implementation strategies identified included staff education, mandated change, the introduction of new recording systems and monitoring practices. These strategies are in keeping with the quality improvement study design used in most of the studies. Notably, the reporting of implementation approaches was generally poor, mostly without use of established implementation frameworks. It was often difficult to extract information about the strategies to apply them to the checklist based on the limited details provided in the published studies. It therefore possible that some additional strategies were used in the included studies, which we were not able to clearly identify, or that we have extrapolated what the studies reported incorrectly into ERIC categories.

These findings offer several insights for advancing implementation science. The successes identified in increasing frailty screening and identification, despite the heterogeneity of screening tools, suggest the feasibility of implementing systematic frailty assessment in hospital settings, providing evidence for the adoptability of such practices. The review also identifies commonly used, relatively simple implementation strategies observed in real‐world settings, such as education, mandates and new systems. While these were not typically framed within theoretical constructs, their application provides a foundation for future research to theoretically ground and systematically evaluate their effectiveness in specific contexts. Additionally, the review highlights the limited range of strategies that have been used in these studies, with opportunity for future studies to explore implementation strategies that have not been used to date.

A critical gap highlighted by this review is the poor reporting quality, underscoring the urgent need for implementation studies in health care to adopt standardised reporting guidelines such as STaRI.^15^ Most of these studies did not identify themselves as implementation studies; only three studies had ‘implementation’ in their title, while 12 studies reported this detail in their abstract. No study referenced the STaRI Statement or checklist however, four of the studies were published prior to 2017 when the STaRI statement was published.^15^ Only three studies used theoretical models to guide study design and implementation, with one using RE‐AIM^20^ and two using the Model for Improvement.^45^ The lack of detail significantly hinders the replicability and scalability of successful interventions, which are core concerns within implementation science.^46^ Furthermore, the reliance on quasi‐experimental designs emphasises the necessity for more robust methodologies, including hybrid effectiveness‐implementation designs.^17^ These designs would enable stronger causal inferences regarding both the effectiveness of interventions and the impact of implementation strategies, thereby contributing to the advancement of implementation science in this area.

The limited attention paid to cultural and organisational context and the lack of process evaluations also point to a significant gap in understanding how and why interventions have succeeded or failed in these studies. Organisational culture and leadership models are key components of successful interventions, which must be understood. There are many reasons that these factors may not be assessed, including the additional time and resources it takes to measure these factors. Future research should prioritise incorporating these elements to enhance the contextualisation and understanding of the mechanisms of action within implementation efforts. The use of frameworks such as Promoting Action on Research Implementation in Health Services (PARIHS)^47^ and Theoretical Domains Framework (TDF)^48^ would assist authors to identify these key features.

Additionally, the absence of consumer engagement in the design and the lack of patient‐centred outcome measures identify crucial areas for future implementation research. Although patient engagement in health‐care research has increased in recent years, there remains a need for active consumer participation in all stages of research.^49^ There were no studies in the review that collected quality of life or patient‐reported outcome measures. This omission is likely attributable to the design of the included studies, as hospital‐based quality improvement work traditionally emphasises process or quantitative outcomes such as length of stay or readmission rates. Quality‐of‐life measures may also not have been considered as they may be less sensitive to change over a short‐term study period and more time‐consuming to collect. Additionally, when populations have a higher likelihood of cognitive impairment, the use of proxy respondents becomes necessary, further complicating the collection and reliability of such measures. Patient reported outcomes measures are, however, an important tool for assessing outcomes from the patient's perspective and give valuable insights into implementation research studies.^50^ Understanding patient perspectives and the impact on their reported outcomes is vital for ensuring the acceptability and appropriateness of frailty interventions.^51^

Finally, the limited explicit use of implementation frameworks by the individual studies, such as ERIC, Consolidated Framework for Implementation Research (CFIR) or RE‐AIM, restricts the ability to systematically categorise and compare implementation strategies across different studies.^52^ Future research should proactively utilise such frameworks to build a more detailed, transferrable and robust evidence base in this area.

### 4.1 Strengths and limitations of this review

To our knowledge, this is the first systematic review of implementation strategies for frailty interventions in hospital settings. The studies included were small and the study designs used resulted in high risk of bias. Only full‐text articles were included in the literature review, and only those available in English. Consequently, it is possible that some relevant studies were missed, including from non‐English‐speaking countries with diverse health‐care settings, limiting the generalisability of the findings. We also restricted our search to published or grey literature in peer‐reviewed journals or online. This may present publication bias with an over‐emphasis of studies with positive findings and the potential omission of studies where implementation has not been successful.

Most of the included studies were conducted in the United States, which is consistent with its leading position in global research output for both health care and frailty research.^53^ However, it is important to consider that the United States has a health system structure that differs significantly from many other countries, potentially limiting the generalisability of findings to health‐care settings outside the United States. Additionally, this review did not identify any studies originating from developing countries, where implementation of frailty interventions in hospital settings may differ substantially due to variations in health‐care infrastructure, resources and clinical practices.

Most of the studies did not use a theoretical model to guide implementation or provide sufficient detail on the implementation methods. Categorising implementation strategies used in the included studies to ERIC categories was sometimes subject to interpretation and therefore may not be entirely accurate. The heterogeneity of the interventions implemented, clinical outcomes and implementation strategies used makes it difficult to draw clear conclusions about which specific implementation strategies are most useful for this patient population.

## 5 CONCLUSIONS

This review demonstrates that implementing frailty screening and interventions in hospitals is feasible and highlights commonly used implementation strategies. However, methodological limitations and poor reporting make it difficult to draw firm conclusions about the effectiveness of specific strategies. To advance the implementation of evidence‐based frailty care, future research should prioritise rigorous study designs, comprehensive reporting and the use of implementation science frameworks. The involvement of consumers and the use of patient‐centred outcome measures were also underutilised in the included studies, yet these elements could play an important role in enhancing the effectiveness and relevance of frailty interventions. Ultimately, understanding and effectively executing successful implementation strategies is crucial to improving the care of people with frailty in hospital settings.

## FUNDING INFORMATION

This work was supported by the 2024 Royal Australasian College of Physicians (RACP) Australasian Faculty of Rehabilitation Medicine (AFRM) Research Entry Scholarship.

## CONFLICT OF INTEREST STATEMENT

No conflicts of interest declared.

## Supporting information


Data S1

Data S2

Data S3

## Data Availability

All data generated or analysed during this study are included in this published article [and its supplementary information files].
