# Supplementary material for: Implementing frailty interventions in hospitals: A systematic review of strategies and outcomes
Source: Australas J Ageing. 2025 Jun 24;44(2):e70060. doi: 10.1111/ajag.70060 (PMC12186596; doi:10.1111/ajag.70060)
Supplement: Supplementary file 2 — Data S2 [file AJAG-44-0-s003.docx]

**Supplementary File 2:** MEDLINE Search Strategy

Ovid MEDLINE(R) and Epub Ahead of Print, In-Process, In-Data-Review & Other Non-Indexed Citations, Daily and Versions <1946 to September 09, 2022>

| Step | Search terms | Records |
| --- | --- | --- |
| 1 | Frailty/ or Frail Elderly/ | 18456 |
| 2 | Health services for the Aged/ | 18152 |
| 3 | frail*.tw. | 30390 |
| 4 | Implementation Science/ or Evidence-Based Medicine/ or Evidence-Based practice/ | 88372 |
| 5 | ("knowledge translation" or "knowledge transfer" or "implementation" or "research translation" or "research transfer").mp. or "evidence translation".ti,ab. [mp=title, book title, abstract, original title, name of substance word, subject heading word, floating sub-heading word, keyword heading word, organism supplementary concept word, protocol supplementary concept word, rare disease supplementary concept word, unique identifier, synonyms] | 326728 |
| 6 | "quality improvement".ti,ab. | 44017 |
| 7 | hospital*.mp. or acute.ti,ab. [mp=title, book title, abstract, original title, name of substance word, subject heading word, floating sub-heading word, keyword heading word, organism supplementary concept word, protocol supplementary concept word, rare disease supplementary concept word, unique identifier, synonyms] | 2999696 |
| 8 | Hospitals/ | 93885 |
| 9 | 1 or 2 or 3 | 51901 |
| 10 | 4 or 5 or 6 | 440582 |
| 11 | 7 or 8 | 2999696 |
| 12 | 9 and 10 and 11 | 870 |
