# Supplementary material for: Implementing frailty interventions in hospitals: A systematic review of strategies and outcomes
Source: Australas J Ageing. 2025 Jun 24;44(2):e70060. doi: 10.1111/ajag.70060 (PMC12186596; doi:10.1111/ajag.70060)
Supplement: Supplementary file 3 — Data S3 [file AJAG-44-0-s001.docx]

**Supplementary File 3:** Risk of Bias Summary

**Pre-Post Studies with No Control Group (Clinical interventions) ***

| **Study Author (Date)** | **Were eligibility / selection criteria for the study population pre-specified and clearly described?** | **Were all eligible participants that met the prespecified entry criteria enrolled?** | **Was the sample size sufficiently large to provide confidence in the findings?** | **Was the intervention clearly described and delivered consistently across the study population?** | **Were the clinical outcome measures pre-specified, clearly defined, valid, reliable, and assessed consistently across all study participants?** | **Were the people assessing the participant outcomes blinded to the participants’ exposures /interventions?** | **Was the loss to follow-up of participants after baseline 20% or less?** | **Did the statistical methods examine changes in clinical outcome measures from before to after the intervention?** |
| --- | --- | --- | --- | --- | --- | --- | --- | --- |
| Bakker (2014) | Yes | Yes | Yes | No | Yes | No | No | Yes |
| Bryant (2019) | Yes | No | No | Unclear | Yes | No | Yes | Yes |
| Chen (2014) | Yes | Yes | No | Yes | Yes | No | Yes | Yes |
| Ehrlich (2022) | Yes | No | Unclear | No | Yes | No | Yes | Yes |
| Engelhardt (2018) | Yes | Yes | No | Yes | Yes | Unclear | No | Yes |
| Ernst (2014) | Yes | Yes | No | Yes | Unclear | Unclear | No | Yes |
| Fritsche (2023) | Yes | No | Unclear | Yes | Yes | Unclear | N/A | Yes |
| Hall (2018) | Yes | Yes | Yes | Yes | Yes | Unclear | No | Yes |
| Hall (2022) | No | Unclear | Unclear | No | No | Unclear | Unclear | No |
| Heim (2016) | Yes | Yes | Unclear | Yes | Yes | No | No | Yes |
| Keiser (2023) | Yes | No | Unclear | Yes | Yes | N/A | N/A | Yes |
| McGrath (2019) | Yes | No | Unclear | Unclear | Yes | No | Yes | Yes |
| Street (2023) | Yes | Yes | Unclear | No | Yes | Unclear | Yes | Yes |
| Wilson (2021) | Yes | No | No | No | Yes | No | No | Yes |

* Adapted from the National Institute of Health (NIH) quality assessment tool for before-after (Pre-Post) study with no control group

**Non-randomised controlled trial****

| **Study Author (Date)** | **Was the study described as randomized, a randomized trial, a randomized clinical trial, or an RCT?** | **Were study participants and providers blinded to treatment group assignment?** | **Were the people assessing the outcomes blinded to the participants' group assignments?** | **Were the groups similar at baseline on important characteristics that could affect outcomes?** | **Was the overall drop-out rate from the study at endpoint 20% or lower of the number allocated to treatment?** | **Was there high adherence to the intervention protocols for each treatment group?** | **Were outcomes assessed using valid and reliable measures, implemented consistently across all study participants?** | **Did the authors report that the sample size was sufficiently large to be able to detect a difference in the main outcome between groups with at least 80% power?** | **Were all randomized participants analysed in the group to which they were originally assigned, i.e., did they use an intention-to-treat analysis?** |
| --- | --- | --- | --- | --- | --- | --- | --- | --- | --- |
| Karlekar (2016) | No | No | No | Yes | Yes | No | No | No | Yes |

**Adapted from the National Institute of Health (NIH) quality assessment tool of controlled intervention studies
